# The Role of IL-1 Family Members and Kupffer Cells in Liver Regeneration

**DOI:** 10.1155/2016/6495793

**Published:** 2016-03-22

**Authors:** Quanhui Tan, Jianjun Hu, Xiaolan Yu, Wen Guan, Huili Lu, Yan Yu, Yongsheng Yu, Guoqiang Zang, Zhenghao Tang

**Affiliations:** ^1^Department of Infectious Disease, Shanghai Jiao Tong University Affiliated Sixth People's Hospital, Shanghai 200233, China; ^2^Shanghai Municipality Key Laboratory of Veterinary Biotechnology, School of Agriculture and Biology, Shanghai Jiao Tong University, Shanghai 200240, China; ^3^Laboratory of Regenerative Medicine, School of Pharmacy, Shanghai Jiao Tong University, Shanghai 200240, China

## Abstract

Interleukin-1 (IL-1) family and Kupffer cells are linked with liver regeneration, but their precise roles remain unclear. IL-1 family members are pleiotropic factors with a range of biological roles in liver diseases, inducing hepatitis, cirrhosis, and hepatocellular carcinoma, as well as liver regeneration. Kupffer cells are the main source of IL-1 and IL-1 receptor antagonist (IL-1Ra), the key members of IL-1 family. This systemic review highlights a close association of IL-1 family members and Kupffer cells with liver regeneration, although their specific roles are inconclusive. Moreover, IL-1 members are proposed to induce effects on liver regeneration through Kupffer cells.

## 1. Introduction

The liver has a remarkable ability to regenerate quickly following injury by a variety of factors. Liver regeneration is a highly coordinated process that involves an array of effector cell proliferative responses, including cell-based liver parenchymal cell regeneration and regeneration by stem cells starting to differentiate into hepatocytes and biliary cells. The cells associated with liver regeneration are closely related to the degree of liver damage. Multiple genes and cytokines as well as the liver microenvironment play crucial roles in the dynamic regulation of regeneration via complex mechanisms that are not fully understood at present. Comprehensive analysis of the liver regeneration process may therefore provide a theoretical basis to develop effective therapeutic strategies that facilitate regeneration for clinical early intervention and recovery of liver function.

The mechanism underlying Kupffer cell-mediated secretion of factors that regulate liver regeneration remains to be established. Release of tumor necrosis factor-*α* (TNF-*α*) by Kupffer cells may increase interleukin- (IL-) 6 expression, further inducing liver cell transition from G0 to G1 phase and promoting hepatocyte proliferation [1]. The progress of liver regeneration is reported to be slowed or inhibited upon inactivation of Kupffer cells [2]. In contrast, other studies have shown that inactivated Kupffer cells promote liver regeneration [3]. These conflicting roles of Kupffer cells in liver regeneration are indicative of their involvement in complex signaling mechanisms.

IL-1, mainly secreted by monocytes, macrophages, epithelial cells, synovial cells, and chondrocytes, plays a crucial role in regulating immune and inflammatory responses. Members of IL-1 family are widely involved in various diseases, including cancer and liver failure, as well as liver regeneration. In recent years, accumulating studies have reported that IL-1 type I receptor (IL-1RI) signaling pathway affects liver regeneration to a certain extent, although the precise mechanisms are yet to be determined.

In this systemic review, the critical roles of IL-1 family members and Kupffer cells in liver regeneration are highlighted.

## 2. IL-1 Family Members

Members of IL-1 family include IL-1F1 (IL-1*α*), IL-1F2 (IL-1*β*), and IL-1F3 (IL-1R*α*). IL-1*α* and IL-1*β* were initially identified during research on immune defense. Both molecules function as proinflammatory cytokines, while IL-1R*α* is an anti-inflammatory cytokine that blocks the pathway downstream of IL-1 signaling and maintains homeostasis by competitively binding IL-1*α* and IL-1*β* receptors [4, 5]. IL-1 is a pleiotropic factor that plays crucial roles in a range of biological processes, such as autoimmune diseases, allergies, infectious diseases, and cardiopulmonary and other human disorders [6]. Earlier studies have shown IL-1 can induce the expression of various genes and related cytokines [7], including acute-phase proteins, tissue remodeling enzymes, and adhesion molecules, in different cell types [8].

Two forms of IL-1, IL-1*α* and IL-1*β*, exist* in vivo*. The majority of cells, in particular epithelial cells, constitutively express pro-IL-1*α*, which is crucial for cell differentiation and cellular immune responses. Expression of IL-1 is significantly increased along with inflammation responses [9]. In contrast, IL-1*β* expression is extremely limited in normal physiological conditions. Under conditions of inflammatory stimulation, IL-1*β* is secreted and released into the bloodstream where it exerts effects on other cells [5, 10].

IL-1R*α* is a natural endogenous IL-1-specific inhibitor produced by several cell types, including monocytes, macrophages, keratinocytes, and endothelial cells. IL-1R*α* is a receptor antagonist with no agonist effect that binds specifically to the IL-1 receptor. IL-1R*α* blocks IL-1 downstream signaling pathways by competing with IL-1R binding placeholder in a dose-dependent manner [5, 10].

IL-1 and IL-1Ra have two coreceptors, specifically, type I IL-1 and type II IL-1 receptors. IL-1RI binds to IL-1*α* or IL-1*β*, further promoting signal transduction pathway activation and various reactions within cells. IL-1RII has been classified as an inert receptor, since signals could not be effectively transmitted after combination with IL-1 [10, 11]. Type II receptor binds competitively to IL-1 or IL-1*β* and suppresses the IL-1 intracellular signaling pathway [5, 6].

## 3. Liver Regeneration

The process of liver regeneration involves complex interactions between hepatocytes and liver nonparenchymal cells, including Kupffer, sinusoidal endothelial, hepatic stellate, and stem cells [12]. Nonparenchymal cells are crucial for signal transduction as well as synthesis and secretion of cytokines and growth factors, which play complex regulatory roles in the process of liver regeneration [13–18]. Accordingly, regulation of nonparenchymal cells in liver regeneration is one of the current hot topics of research interest [19, 20]. Overall, liver regeneration is divided into three stages: triggering, proliferation and growth, and termination. Under physiological conditions, liver cells are in the resting state of the G0 phase. Once liver regeneration is triggered, the mitogen shows significant expression, prompting entry of cells into the G1 phase (G0/G1 transition). This conversion is highly dependent on a number of key cytokines, such as IL-6 and TNF-*α* [21]. Previous studies have shown that at the trigger stage more than 40% of early gene expression is IL-6 dependent. Upon knockout of IL-6, acute liver failure was reported in 70% mice. Moreover, IL-6 induces a significant increase in nonparenchymal cells that produce hepatocyte growth factor and further promotes liver cell proliferation through the signal transducer and activator of transcription 3 signaling pathway [22–25]. After G0/G1 conversion, the majority of the remaining hepatocytes (95%) enter the growth phase. Growth factor signaling pathways, particularly the hepatocyte growth factor (HGF)/C-met (HGF receptor) pathway, play a major role during this stage. Moreover, growth factor signaling pathways begin to play a role in the late G1 phase and stimulate hepatocyte-inducible expression of cell cycle regulatory proteins, such as cyclin D1 and P21, by pushing through the cell cycle G1 restriction point and enter S phase [26, 27]. The liver cell number during the regeneration process is increased mainly through the IL-6/signal transducer and activator of transcription 3 (STAT-3) pathway while cell volume is predominantly increased through the phosphoinositide 3-kinase/3-phosphoinositide-dependent protein kinase 1/protein kinase B pathway [12]. In the terminal phase of liver regeneration, a number of negative regulators of cell proliferation and cytokine expression, such as transforming growth factor *β*1, are upregulated and play an important role in preventing excessive regeneration [28].

## 4. IL-1 Family Members and Liver Regeneration

Macrophages (including Kupffer cells and their predecessor) are the main source of IL-1 and IL-1Ra, key members of the IL-1 family. The family includes two major active ligands, IL-1*α* and IL-1*β* (both active forms of IL-1), an antagonist ligand, IL-1Ra, and type I (IL-1RI) and type II (IL-1RII) receptors. IL-1RI is an active receptor, which can transduce signals among cells and is expressed in almost all cells, especially activated nonparenchymal cells. IL-1, IL-1Ra, and IL-1RI constitute a highly conserved biological IL-1 system that is widely involved in various inflammatory and stress responses [29].

Earlier studies have disclosed that the IL-1RI signaling pathway plays important roles in liver regeneration after acute liver failure and partial hepatectomy, although the exact mechanisms remain to be established. In recent years, increasing research has focused on the effects of regulating the signal transduction pathway of IL-1RI on liver regeneration. Ma et al. found that IL-1*β* siRNA adenovirus combined with mesenchymal stem cell reduced the inflammatory levels, prevented liver failure, promoted liver regeneration, and increased survival rates in CCl_4_-induced acute liver failure mice, indicating inhibiting role of IL-1*β* in liver regeneration after acute liver failure [30]. Wang et al. showed that recombinant human augmenter of liver regeneration promoted liver regeneration, attenuated liver injury, increased overall survival, and suppressed immunological responses in d-galactosamine (GaIN) induced acute liver failure rats, along with significantly reduced IL-1*β* of serum and ascites, suggesting an important role of IL-1*β* in liver regeneration [31]. Zhu et al. detected the role of recombinant human interleukin-1 receptor antagonist (rhIL-1Ra) in liver regeneration and found that subcutaneous injection of rhIL-1Ra decreased the centrilobular necrotic areas and increased hepatocyte proliferation and survival benefit of CCl_4_-induced acute liver failure mice and demonstrated that rhIL-1Ra administration ameliorated the histological damage and accelerated the regeneration and recovery process of the liver [32]. These findings support a positive regulatory function of IL-1Ra and a negative regulatory function of IL-1*β* in liver regeneration after acute liver failure.

In another category of liver regeneration after partial hepatectomy, Boermeester et al. showed that, using IL-1Ra to antagonize 2/3 partial hepatectomy (PH) rat-derived IL-1, residual liver inflammation was significantly reduced and the number of PCNA-positive cells was significantly increased, indicating that IL-1-mediated liver inflammation is an important factor in 2/3 PH rat liver failure [33]. Sgroi and colleagues also reported that rhIL-1Ra plays a significant promoting role in the liver regeneration rate in mice at the early (24 h) stages after 70% PH [34]. These findings collectively support a positive regulatory function of IL-1Ra in liver regeneration, although the precise mechanisms are yet to be ascertained.

According to the known mechanism, IL-1 family for IL-1Ra and IL-1RI knockout in liver regeneration model should theoretically have similar biological effects, and the meaning of the highlighted sentence appears unclear for interpretation. To examine this theory, we compared 1/3 PH liver regeneration among IL-1RI (KO) and wild-type mice. Preliminary results showed that in the early stages (24 h), liver regeneration speed was significantly slower in IL-1RI-KO than wild-type mice. Nevertheless, at the later stages (96 h), the extent of liver regeneration in IL-1RI-KO and wild-type mice gradually converged. Liver regeneration was restored to the preoperative level at 168 h. The conflicting theoretical predictions and experimental results obtained to date highlight the extremely complex nature of the IL-1RI pathways that regulate liver regeneration. In summary, we infer that inhibition of the IL-1RI signaling pathway in liver cells can contribute to liver regeneration. Under physiological conditions, the IL-1RI signaling pathway remains in the lower activation state, which may be beneficial in rapid secretion of triggering cytokines for liver regeneration after PH.

A recombinant human IL-1 receptor antagonist has been approved by Food and Drug Association (FDA) as conventional therapy for refractory rheumatoid arthritis and the complete indications of phase III clinical studies documented. Earlier studies by our group disclosed that IL-1Ra reduces hepatocyte death and accelerates liver cell proliferation. In our experiments, rhIL-1Ra significantly improved the overall survival rate of mice subjected to paracetamol- (acetaminophen-) induced acute liver failure. Furthermore, our data confirmed that the critical role of rhIL-1Ra in protecting against acute liver failure is dependent on reducing hepatic cell apoptosis to a significant extent as well as promoting liver regeneration [35]. Sgroi et al. showed that IL-1Ra accelerates cell proliferation at the early stage of liver regeneration in PH mice by reducing inflammation pressure [34]. Zhu and colleagues demonstrated that rhIL-1Ra has the ability to reduce liver cell damage and promote liver regeneration [32]. The group of Petrasek reported that IL-1RA can ameliorate inflammasome-dependent alcoholic steatohepatitis in mice [28]. The researchers further suggested that Casp-1 expressed in Kupffer cells is involved in alcohol-induced liver inflammation, steatosis, and injury. However, their results did not support a significant pathogenic role for Casp-1 in the development of alcoholic liver disease. Sgroi et al. showed that overexpression of IL-1Ra results in inhibition of liver failure and reduction of mortality in rats with fulminant hepatic failure [34]. Other researchers found that IL-1*β* markedly stimulates nitric oxide formation of primary cultured rat hepatocytes in the absence of lipopolysaccharide [36].

These studies collectively highlight a critical role of the IL-1RI pathway in the process of liver regeneration. However, further research is needed to establish the underlying regulatory mechanisms. Based on the above conclusions, we infer that Kupffer cells play a negative regulatory role in liver regeneration after PH, while other nonparenchymal cells play a positive regulatory role. The physiological balance between these two factors determines the final outcome, that is, compensatory liver regeneration or acute liver failure. Kupffer cells serve as key targets of the IL-1RI pathway while the combination of IL-1Ra and IL-1RI inhibits downstream signaling pathways and suppresses the negative regulatory role of Kupffer cells in liver regeneration. Other nonparenchymal cells may additionally secrete more positive regulators during liver regeneration process and ultimately facilitate acceleration of liver regeneration. Moreover, the effects of the IL-1RI pathway on liver regeneration are mainly exerted in the early stages.

In summary, clarification of the mechanisms underlying the involvement of the IL-1RI pathway in liver regeneration may aid in the development of new targets and strategies in liver regeneration therapy.

## 5. Kupffer Cells and Liver Regeneration

Kupffer cells account for 80%–90% of all macrophages and 35% of nonparenchymal cells in the liver. Partial resection of residual liver Kupffer cells can promote intestinal endotoxemia and secretion of large amounts of cytokines, which are considered to play important regulatory roles in acute liver failure and liver regeneration. Kupffer cells have been characterized as immunoregulatory cells that are critical in liver regeneration. However, their precise function in liver regeneration is under dispute. Some studies indicate that liver regeneration requires several steps, including the innate immune response, in particular, IL-6 and TNF-*α* production by Kupffer cells, although the activation process remains unknown. Other researchers have shown that Kupffer cells produce important cytokines that induce cell proliferation after hepatectomy [37].

The role of Kupffer cells in liver regeneration was further investigated by interference with their function. Boulton et al. reported that the overall effect of Kupffer cell inhibition was improvement in the liver cell proliferation rate, whether after PH or at the resting state [38]. Additionally, Kupffer cells were shown to have a stimulatory effect on liver regeneration mainly through activation of NF-*κ*B. Kupffer cells influence cell cycle and proliferation by enhancing the expression of cytokines, such as TNF-*α* and IL-6 [39]. A number of studies have confirmed a protective role of Kupffer cells in liver cells. Murata et al. demonstrated that after PH liver regeneration is delayed in Kupffer cell-depleted mice owing to low expression of TNF-*α* [40]. Another study showed that Kupffer cell depletion with GdCl_3_ enhances liver regeneration and increases overall survival of mice after PH [3]. Amemiya et al. confirmed that Kupffer cells play a key role in liver regeneration after PH. The group observed that Kupffer cell activation and liver regeneration in osteopetrotic mice after macrophage colony-stimulating factor (M-CSF) treatment reach a similar extent to those of mice in their nest [41]. A study of gene chips of Kupffer cells in rats after 2/3 PH by Xu et al. disclosed that the genes involved in cell division declined from 2–12 h to significantly lower levels than those before surgery. Gene expression peaked at 24 h and then declined gradually but was still significantly higher than that at the level of resting, even at the end of liver regeneration (168 h) [42]. In contrast, Jiang et al. demonstrated that after PH liver cells and expression of cell division genes are slightly increased within 2 h, peak at 24 h, and then gradually decline [43]. Both studies demonstrated a backward linkage between Kupffer cells and proliferation at the early stages of liver regeneration.

Research to date has implicated a dual role of Kupffer cells in liver regeneration. Acute liver failure was reported to promote liver regeneration induced by Kupffer cells and the rate of regeneration was associated with the level of acute liver failure [44]. Depletion of Kupffer cells improved the survival rates of liver transplantation, reduced liver damage, and promoted regeneration by IL-6 [45]. Suzuki et al. indicated that inhibiting Kupffer cell function protects mice from endotoxin-induced liver injury after hepatectomy [46]. Kupffer cell function in liver regeneration may be different in the early start-up and late proliferative phases [47]. Recent studies suggest that Kupffer cell depletion affects vasoactivity and liver blood perfusion. Kupffer cell-dependent molecular mechanisms are proposed to be involved in vasoactive mediation during the liver generation progress, and maintenance of vascular shear stress may be the trigger of hepatocyte proliferation [7]. ATP released from hepatocytes and Kupffer cells under mechanical stress in liver resection can promote liver regeneration in rats. Yang et al. suggested that the functional integrity of Kupffer cells plays an important role in liver regeneration, since TNF-*α*, IL-6, and other cytokines are released by activated Kupffer cells [48]. These cytokines initiate the regeneration process to a certain extent through the NF-*κ*B and STAT3 pathways. However, recent studies have shown that the proposed process involves intercellular interactions between leukocytes and Kupffer cells, leading to direct activation of Kupffer cells [49]. Interactions between complement activation and leukocyte Kupffer cells were additionally shown to promote liver cell regeneration. Rai and coworkers found that preoperative use of gadolinium chloride to eliminate Kupffer cell function significantly accelerates the liver regeneration speed in rats.

Based on these findings, we conclude that Kupffer cells play a major role in inhibition of liver cell proliferation during the process of regeneration. These cells control liver regeneration to a reasonable limit after PH while promoting acute liver failure in lethal PH. There may be both a synergistic and antagonistic balance between Kupffer cells and nonparenchymal cells during liver regeneration. When Kupffer cells are dysfunctional, other nonparenchymal cells can secrete important cytokines, such as IL-6 and TNF-*α*, while in the coexistence state other nonparenchymal cells may suppress the regulatory role of Kupffer cells in liver regeneration.

The specific mechanisms of Kupffer cell action in liver regeneration remain to be elucidated. The group of Luo showed that Kupffer cells contribute to liver regeneration by inhibiting apoptosis through the IL-6/p-STAT3 activation pathway [50]. Rai and coworkers suggested that IL-10 production by Kupffer cells is an important mechanism that suppresses TNF production during liver regeneration [51]. Hepatocytes are “primed” mainly by Kupffer cells via cytokines (IL-6 and TNF-*α*), following which “proliferation” and “cell growth” are induced by stimulation of cytokines and growth factors (HGF and TNF-*α*) [12]. Kupffer cells are additionally involved in the postnecrotic proliferative liver states and play a stimulatory role in liver regeneration by enhancing HGF expression via non-TNF-*α* mediated mechanisms [52, 53].

## 6. Conclusions

IL-1 family members and Kupffer cells have a synergistic effect in inducing liver regeneration. IL-1Ra secreted by Kupffer cells and some other immune cells plays a crucial role in promoting liver regeneration. There are also some cytokines produced by Kupffer cells, such as IL-6, IL-1*β*, and TNF-*α*, which were reported inhibiting the process of liver regeneration. In addition, Kupffer cells are often influenced in liver microenvironment, which further alters their roles in liver regeneration. Therefore the precise associations of IL-1 family members, Kupffer cells, and liver regeneration are inconclusive and the further investigation should focus on the precise mechanism under which IL-1 family members and Kupffer cells promote or inhibit liver regeneration.
